# Supplementary material for: Pharmacological targeting of host chaperones protects from pertussis toxin in vitro and in vivo
Source: Sci Rep. 2021 Mar 8;11:5429. doi: 10.1038/s41598-021-84817-2 (PMC7940712; doi:10.1038/s41598-021-84817-2)
Supplement: Supplementary file 1 — Supplementary figures. [file 41598_2021_84817_MOESM1_ESM.docx]

**Supplementary Information**

**Pharmacological targeting of host chaperones protects from pertussis toxin *in vitro* and *in vivo***

**Katharina Ernst^1#^*, Ann-Katrin Mittler^1#^, Veronika Winkelmann^2^, Carolin Kling^1^, Nina Eberhardt^1^, Anna Anastasia^1^, Michael Sonnabend^1^, Robin Lochbaum^2^, Jan Wirsching^1^, Moona Sakari^3^, Arto T. Pulliainen^3^, Ciaran Skerry^4^, Nicholas H. Carbonetti^4^, Manfred Frick^2^ and Holger Barth^1^***

^1^Institute of Pharmacology and Toxicology, University of Ulm Medical Center, Ulm, Germany

^2^Institute of General Physiology, University of Ulm, Ulm, Germany

^3^Institute of Biomedicine, Research Unit for Infection and Immunity, University of Turku, Turku, Finland

^4^Department of Microbiology & Immunology, University of Maryland School of Medicine, Baltimore, MD, USA

Running title: Chaperones facilitate pertussis toxin uptake into cells

*Katharina Ernst: Institute of Pharmacology and Toxicology, University of Ulm Medical Center, Ulm, Germany; katharina.ernst@uni-ulm.de, Tel. +49 731 50065528, Fax. +49 0731 50065502;
Holger Barth: Institute of Pharmacology and Toxicology, University of Ulm Medical Center, Ulm, Germany; holger.barth@uni-ulm.de, Tel. +49 731 50065503, Fax. +49 0731 50065502

^#^These authors contributed equally to this work

**Keywords:** bacterial toxins, intracellular transport, chaperones, pharmacological inhibitors, human primary epithelium, pertussis toxin, Bordetella


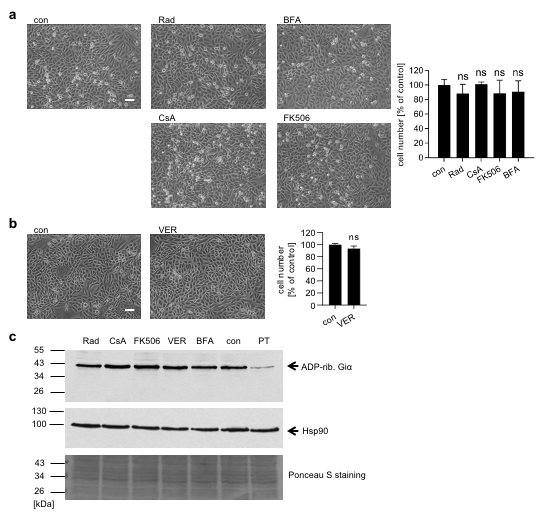


**S1 Figure. a. Effect of Rad, CsA, FK506 and VER on morphology of CHO-K1 cells and ADP-ribosylation of Giα.** CHO-K1 cells were incubated with 20 µM of Rad, CsA, FK506 or BFA **(a)** or 30 µM VER **(b)** for 2 h or left untreated. Medium was removed and cells were further incubated at 37 °C in fresh medium without inhibitor. Pictures were taken after 18 h. Scale bar = 50 µm. **c.** CHO-K1 cells were incubated with 20 µM of Rad, CsA, FK506 or BFA or left untreated for control (con). For further control, cells were treated with 10 ng/ml PT. After 4 h, cells were washed, lysed and incubated with rPTxS1 and biotin-labeled NAD+ at RT. Then, samples were subjected SDS-PAGE and ADP-ribosylated i.e., biotin-labeled Giα was detected in Western Blot analysis. Protein loading was assessed by Ponceau S staining. One representative result is shown (n = 3). For uncropped blots see supplemental figure S14.


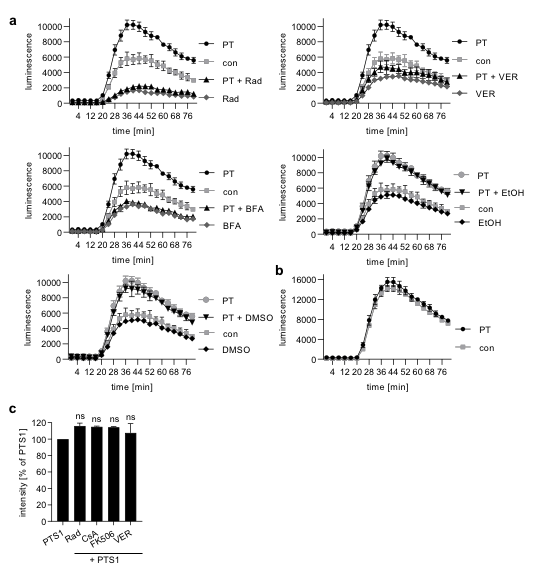


**S2 Figure. a. Effect of Rad, CsA, FK506 and VER on intracellular cAMP levels.** iGIST sensor cells (HEK293 cells expressing SSTR2 and luminescent cAMP probe) were incubated with Rad, VER or BFA (5 µM) or the corresponding solvents (ethanol (EtOH), DMSO) or left untreated for control for 30 min. 500 ng/ml PT were added for 3 h and afterwards inducing medium was added to start the luminescence reaction. A baseline was recorded for 15 min. Then, cells were treated with forskolin to activate adenylate cyclase and with octreotide to activate SSTR2. Luminescence was recorded for 1 h. Values are given as mean ± SD. Results from one representative experiment are shown. **b.** iGIST sensor cells were treated with 500 ng/ml PT or left untreated for control. A baseline was recorded for 15 min after addition of inducing medium. Then only forskolin was added and luminescence was recorded for 1 h. Values are given as mean ± SD. Results from one representative experiment are shown. **c. Effect of Rad, CsA, FK506 and VER on enzyme activity *in vitro*.** Post nuclear supernatant (PNS) from CHO-K1 was gained from confluently cultivated CHO-K1 cells which were lysed in ADP-ribosylation buffer (0.1 mM Tris-HCL (pH 7.6), 20 mM DTT and 0.1 µM ATP) and centrifuged at 3,000 g at 4 °C for 5 min. CHO-K1 cell lysates were pre-incubated with 20 µM Rad, CsA, FK506 or 30 µM VER for 30 min or with buffer for control at room temperature. After 30 min, 170 ng PTS1 (Aviva Systems, San Diego, California, USA) and 10 µM biotin-labeled NAD^+^ were added and incubated for 30 min at room temperature. Samples were subjected to SDS-PAGE, blotted and ADP-ribosylated Giα was detected with Strep-POD. Western blot signals were quantified and normalized to loaded protein. Values are given as percent of samples treated with PTS1 only (n = 3, mean ± SD). Significance was tested by one-way ANOVA with Dunnett’s multiple comparison test and refers to samples treated with PTS1 only (ns = not significant).

**
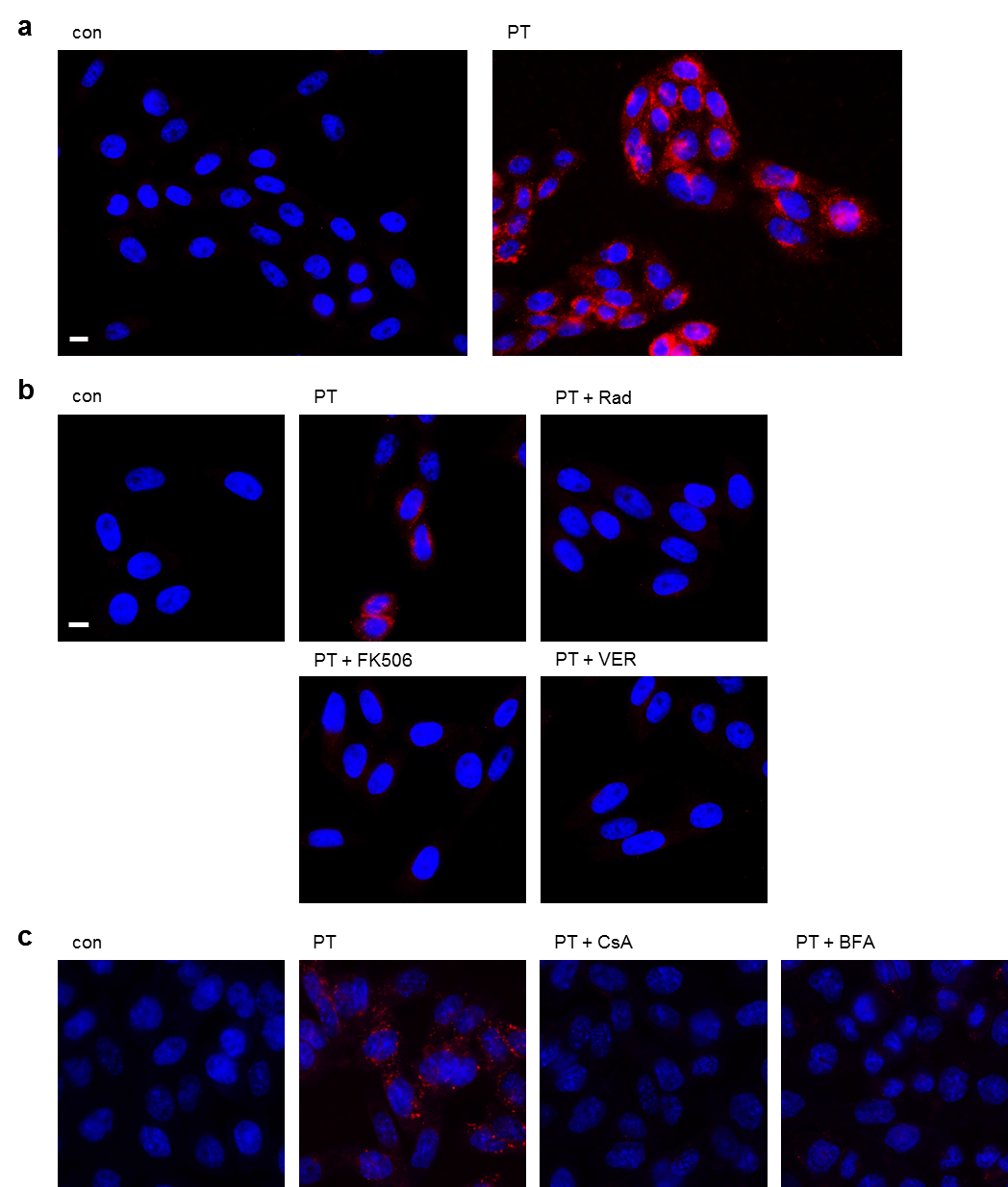
**

**S3 Figure. In the presence of inhibitors of Hsp90/Hsp70 and PPIases less free PTS1 is detected in CHO-K1 cells. a.** CHO-K1 cells were incubated with 1 µg/ml PT or left untreated for control for 24 h. PTS1 was detected by immunofluorescence. Scale bar = 10 µm. **b. and c.** CHO-K1 cells were pre-incubated with CsA, FK506, Rad (20 µM) or VER (30 µM) for 30 min or left untreated for control. 20 µM BFA were used as further control. Then, cells were challenged with 0.4 µg/ml PT. After 3 h, PTS1 was detected by immunofluorescence. Scale bar = 10 µm. Red = PTS1, blue = nucleus (Hoechst staining).


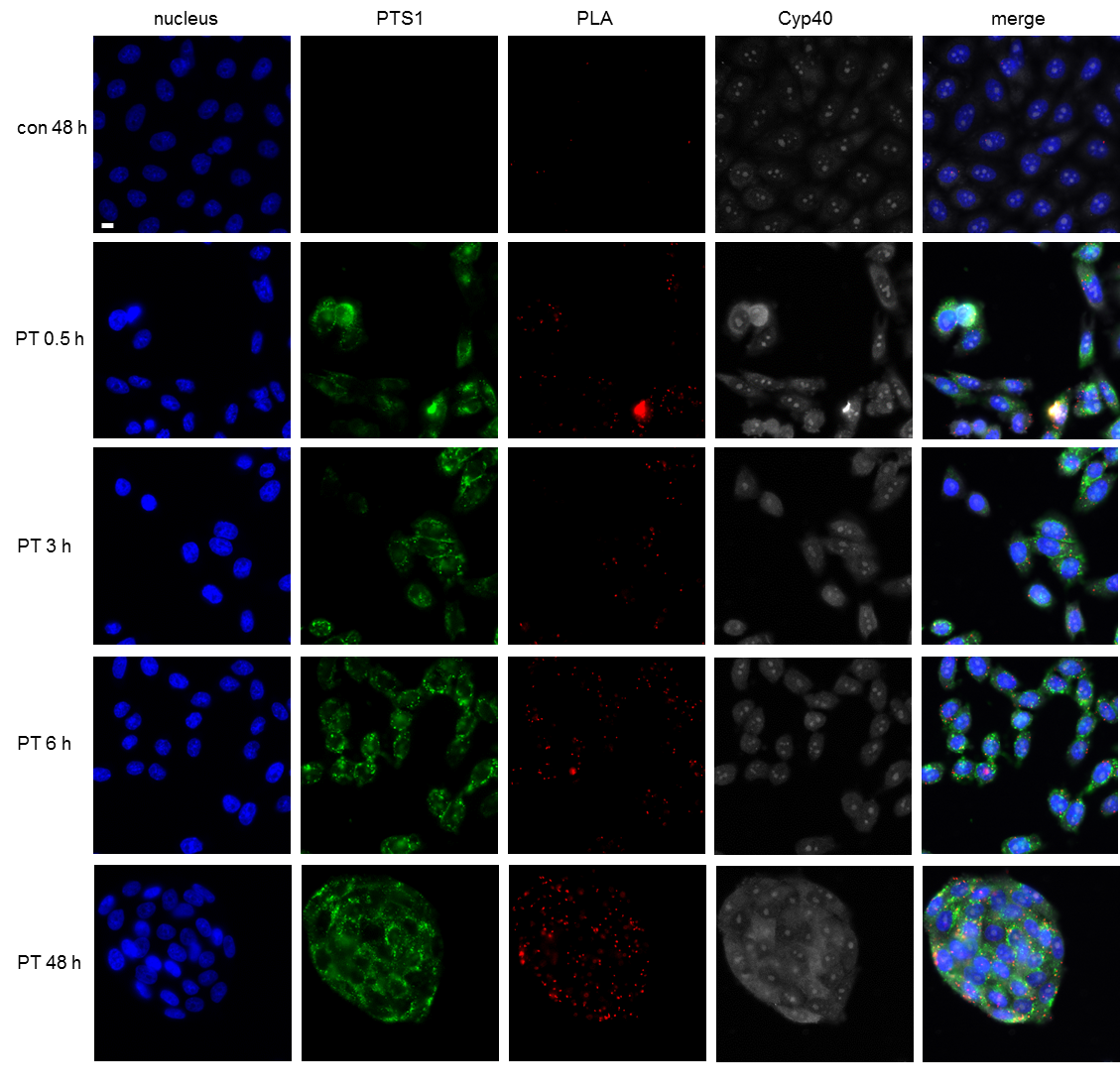


**S4 Figure. Co-staining of PLA with PTS1 and Cyp40 signals.** CHO-K1 cells were incubated on ice with PT (5 µg/ml) for 30 min to enable binding or left untreated for control. After washing, the cells were further incubated for 0.5 h, 3 h, 6 h and 48 h at 37 °C. Cells were fixed and fluorescence-based PLA assay was performed according to the manufacturer´s manual. One representative image is shown for each condition. Scale bar = 10 µm.


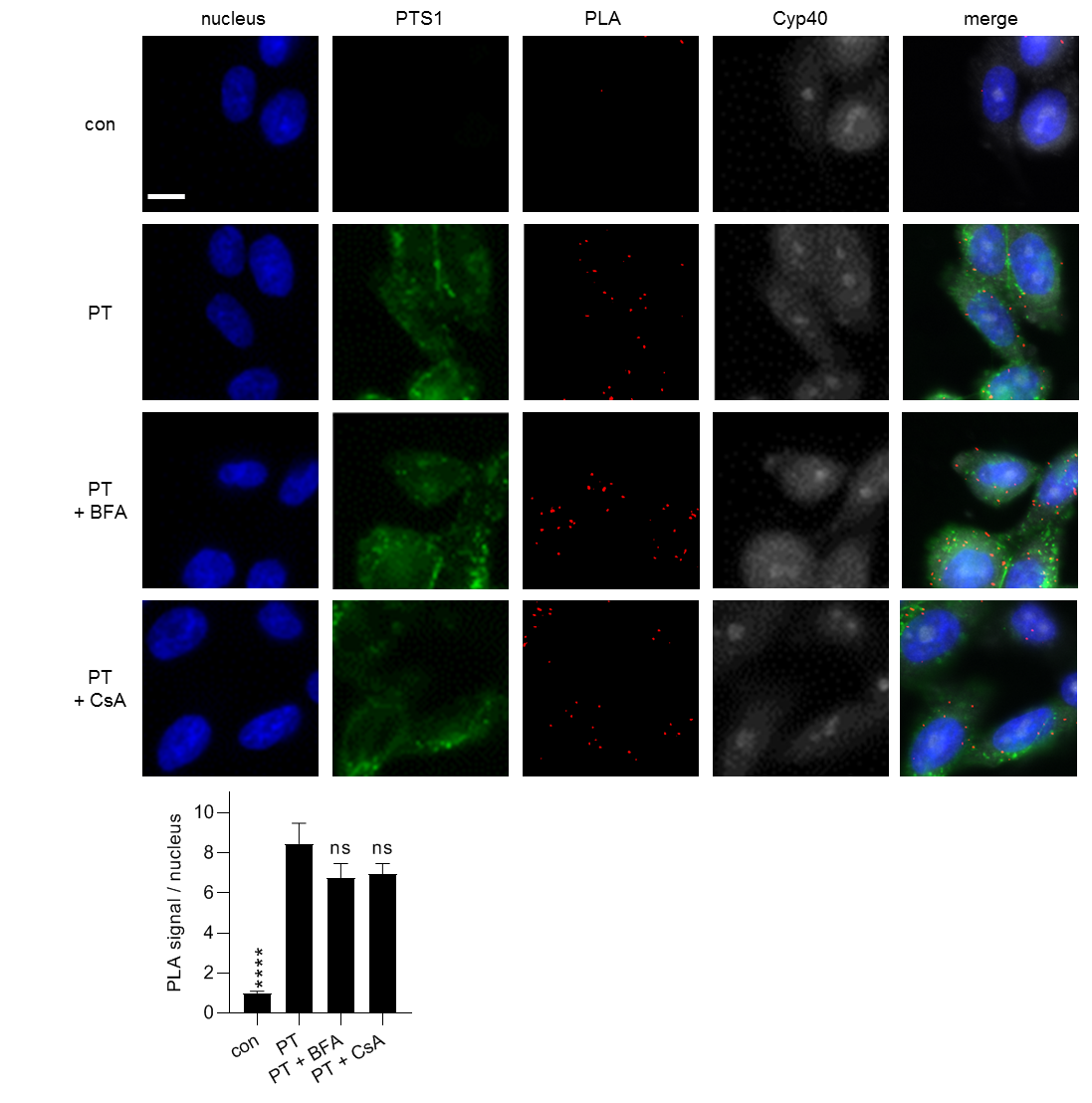


**S5 Figure. Effect of BFA and CsA on interaction of Cyp40 with PTS1 in cells.** CHO-K1 cells were pre-incubated with BFA or CsA (20 µM) for 30 min. Then, PT (5 µg/ml) was added for 3 h at 37 °C or cells were left untreated for control. Cells were fixed and fluorescence-based PLA assay was performed according to the manufacturer´s manual. PLA signals represent one protein interaction event of PTS1 and Cyp40 and were counted from fluorescence pictures (n = 20 pictures per condition from two independent experiments, mean ± SEM) with ImageJ. Values were normalized to the mean of the control samples. Significance was tested by one-way ANOVA with Dunnett’s multiple comparisons test and refers to samples treated with PT only (**** p < 0.0001, ns = not significant). One representative image is shown for each condition. Scale bar = 10 µm.


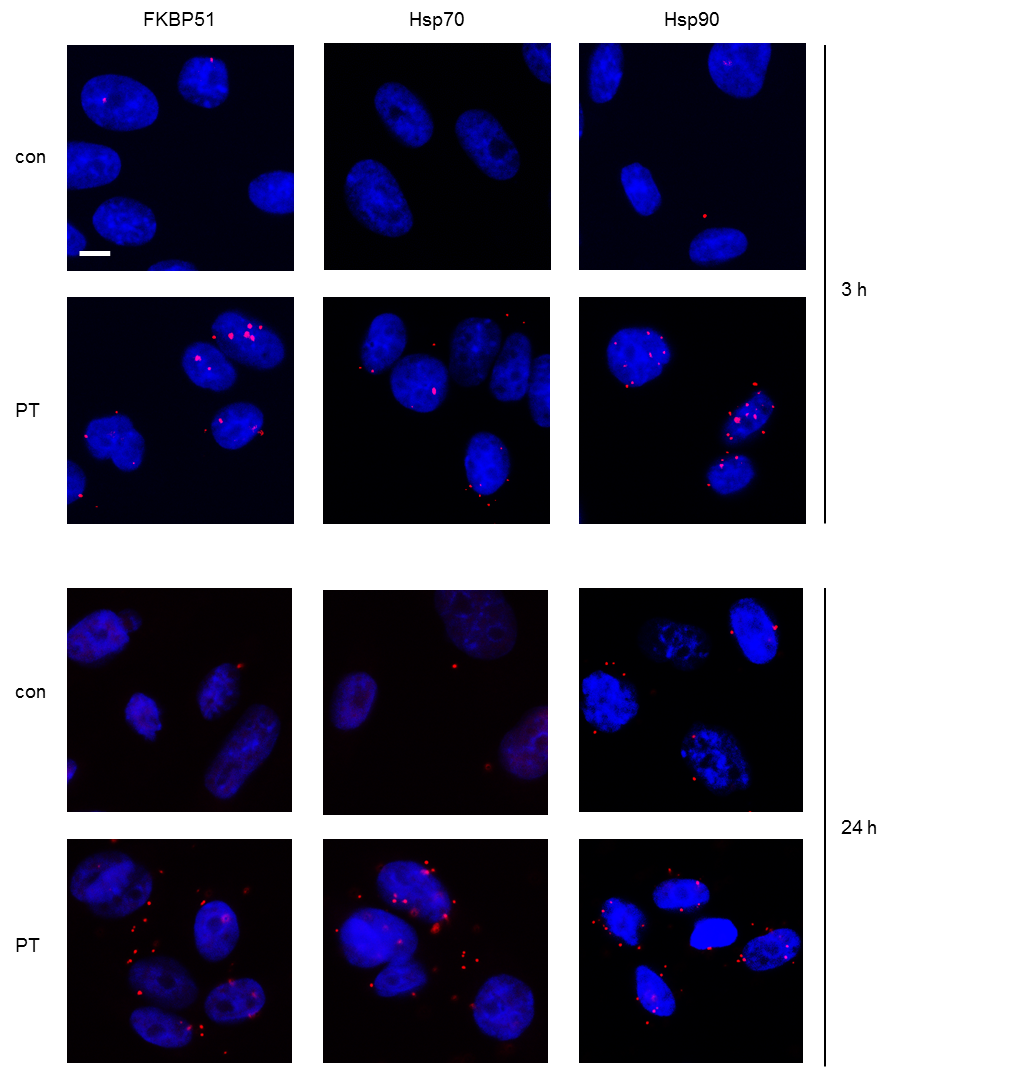


**S6 Figure. FKBP51, Hsp70 and Hsp90 interact with PTS1 in cells.** CHO-K1 cells were treated with PT (1 µg/ml) for 3 h at 37 °C. Then, cells were fixed, and fluorescence-based PLA assay was performed according to the manufacturer´s manual. One representative image per condition is shown (blue = nucleus, red = PLA signal, scale bar = 10 µm).


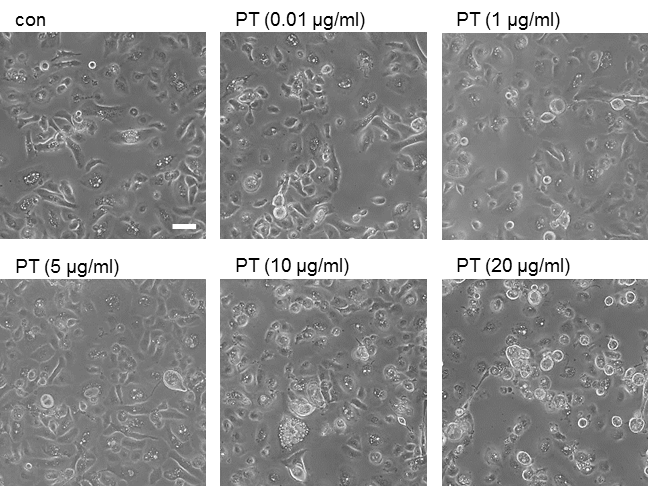


**S7 Figure. Effect of PT on human primary basal cells from bronchial epithelium.** Basal cells were incubated with different concentrations of PT or left untreated for control. Images were obtained after 25 h of PT treatment. Scale bar = 50 µm.


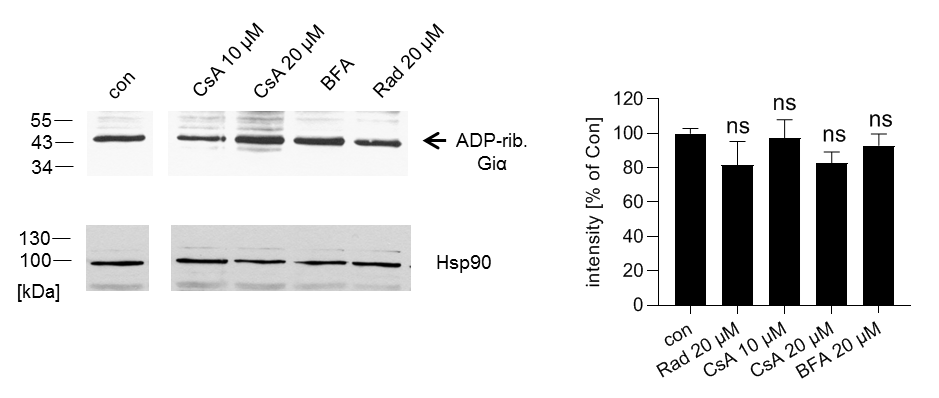


**S8 Figure. Effect of Rad, CsA and BFA on ADP-ribosylation of Giα in basal cells.** Basal cells were incubated with Rad, CsA or BFA (20 µM) for 4.75 h or left untreated for control (con). Subsequently, the ADP-ribosylation status of Giα was determined as described before (Fig 5a). A representative Western blot result is shown. Comparable protein loading was confirmed by Ponceau S (not shown) and Hsp90 staining. Western blot signals were quantified and normalized to protein loading (n = 8 independent experiments, mean ± SEM). Significance was tested by one-way ANOVA with Dunnett’s multiple comparisons test and refers to control samples (ns = not significant). For uncropped blots see supplemental figure S13.


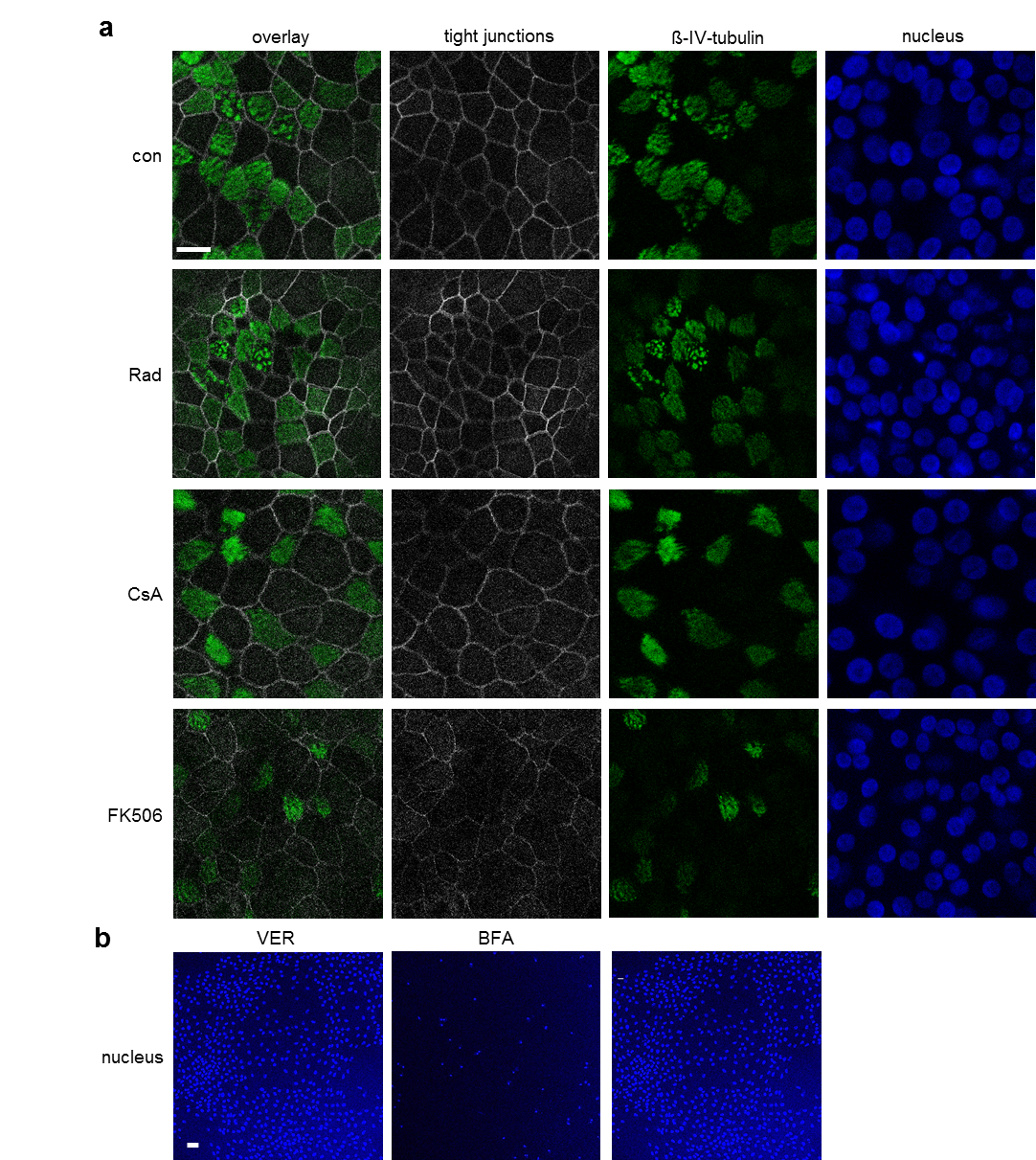


**S9 Figure. Effect of chaperone inhibitors on tight junctions of hBAECs.** hBAECs were incubated with Rad (20 µM), CsA (20 µM), FK506 (20 µM) **(a)**, VER (30 µM) and BfA (20 µM) **(b)** from basolateral side for 72 h or left untreated for control. Cells were fixed with 4 % PFA. For permeabilization and quenching of autofluorescence, cells were treated with 0.2 % saponin. The nuclei were stained with Hoechst33342 (blue) and the tight junctions were stained with ZO-1 (grey). Type IV ß-tubulin (green) was stained with a specific primary antibody and the respective fluorescence-labeled secondary antibody. Pictures were taken with an inverted confocal microscope. Scale bar = 20 µm **(a)**, 40 µm **(b)**.


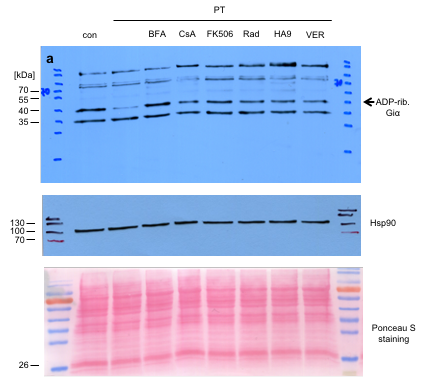


**S10 Figure. Uncropped and unprocessed blots of Fig. 2a**


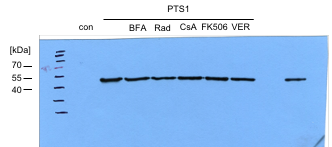


**S11 Figure. Uncropped and unprocessed blot of Fig. 2c**


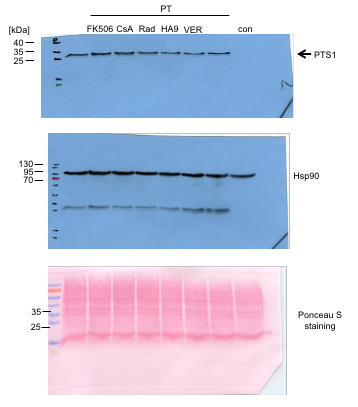


**S12 Figure. Uncropped and unprocessed blots of Fig. 2d**


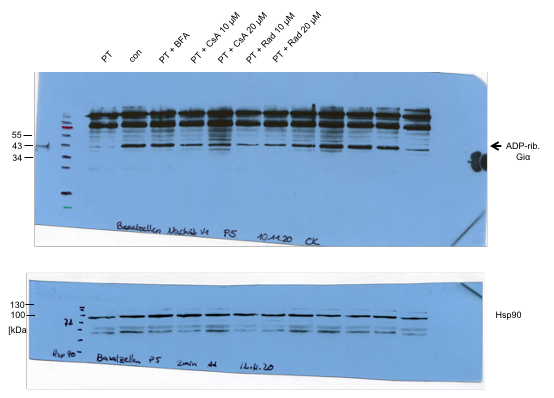


**S13 Figure. Uncropped and unprocessed blots of Fig. 5a**

**
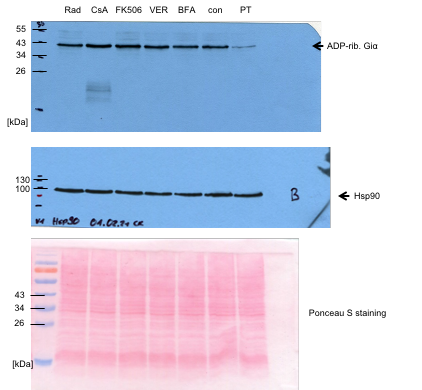
**

**S14 Figure. Uncropped and unprocessed blots of supplemental Fig. 1c**


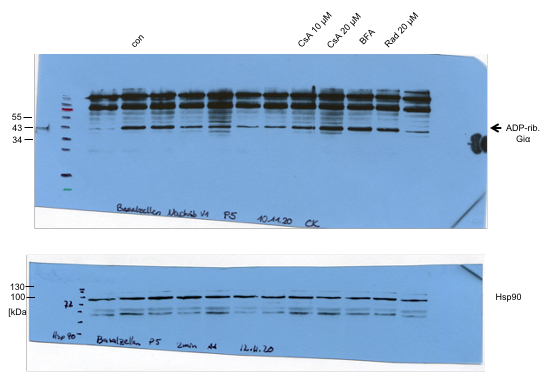


**S15 Figure. Uncropped and unprocessed blots of supplemental Fig. 8**
